# One More Piece in the VACV Ecological Puzzle: Could Peridomestic Rodents Be the Link between Wildlife and Bovine Vaccinia Outbreaks in Brazil?

**DOI:** 10.1371/journal.pone.0007428

**Published:** 2009-10-19

**Authors:** Jônatas S. Abrahão, Maria Isabel M. Guedes, Giliane S. Trindade, Flávio G. Fonseca, Rafael K. Campos, Bruno F. Mota, Zélia I. P. Lobato, André T. Silva-Fernandes, Gisele O. L. Rodrigues, Larissa S. Lima, Paulo C. P. Ferreira, Cláudio A. Bonjardim, Erna G. Kroon

**Affiliations:** 1 Universidade Federal de Minas Gerais, Instituto de Ciências Biológicas, Laboratório de Vírus, Belo Horizonte, Minas Gerais, Brazil; 2 Universidade Federal de Minas Gerais, Instituto de Ciências Biológicas, Laboratório de Virologia Comparada, Belo Horizonte, Minas Gerais, Brazil; 3 Universidade Federal de Minas Gerais, Escola de Veterinária, Laboratório de Vírus, Belo Horizonte, Minas Gerais, Brazil; U.S. Naval Medical Research Center Detachment/Centers for Disease Control, United States of America

## Abstract

**Background:**

Despite the fact that smallpox eradication was declared by the World Health Organization (WHO) in 1980, other poxviruses have emerged and re-emerged, with significant public health and economic impacts. *Vaccinia virus* (VACV), a poxvirus used during the WHO smallpox vaccination campaign, has been involved in zoonotic infections in Brazilian rural areas (Bovine Vaccinia outbreaks – BV), affecting dairy cattle and milkers. Little is known about VACV's natural hosts and its epidemiological and ecological characteristics. Although VACV was isolated and/or serologically detected in Brazilian wild animals, the link between wildlife and farms has not yet been elucidated.

**Methodology/Principal Findings:**

In this study, we describe for the first time, to our knowledge, the isolation of a VACV (Mariana virus - MARV) from a mouse during a BV outbreak. Genetic data, in association with biological assays, showed that this isolate was the same etiological agent causing exanthematic lesions observed in the cattle and human inhabitants of a particular BV-affected area. Phylogenetic analysis grouped MARV with other VACV isolated during BV outbreaks.

**Conclusion/Significance:**

These data provide new biological and epidemiological information on VACV and lead to an interesting question: could peridomestic rodents be the link between wildlife and BV outbreaks?

## Introduction

Thirty years ago, the scientific community celebrated the eradication of smallpox, a highly lethal disease caused by the *Variola virus* (VARV) [1], a member of Family *Poxviridae* [2]. This achievement was the result of a coordinated effort by the World Health Organization (WHO), which promoted a world-wide vaccination campaign during the 1960s and 1970s [3]. The smallpox vaccines used in the WHO campaign were, in fact, *Vaccinia virus* (VACV) strains, a species belonging to the *Orthopoxvirus* (OPV) genus that presents serological cross-reaction with other OPV, including VARV [1], [3]. Despite the immune protection provided by VACV, several cases of adverse manifestations due to vaccination were reported, which led to the suspension of the vaccination campaign after eradication of the disease [4], [5]. Many experts believed that the war against the poxvirus had been won.

However, in recent years, other poxviruses have emerged and re-emerged, causing exanthematic infections in humans and domestic animals, both in rural and urban areas. These zoonotic diseases are mainly caused by OPV species, such as (*i*) *Cowpox virus* (CPXV) in Europe [6]; (*ii*) *Monkeypox virus* (MPXV), which occurs naturally in Africa and was recently introduced in the USA [7]; and (*iii*) *Vaccinia virus* (VACV) in Asia and South America [8]–[10]. The host range of zoonotic OPV remains under investigation, and some naturally infected mammalian species have been described. Serological and molecular approaches have shown that CPXV persists in bank voles, field voles and wood and house mice, while squirrels seem to be the main natural reservoir of MPXV [11]. Despite the historical importance of VACV, there are few data about the origins, natural reservoirs and mechanisms by which the virus persists in nature [12]–[14]. Although some authors believed that VACV vaccine strains could have spread from humans to domestic animals and adapted to the rural environment [8], recent studies have suggested an independent origin for South American VACV isolates, distinct from the vaccine strains used on this continent during the WHO campaign [13], [14].

Today, VACV infections affecting dairy cattle and milkers, mainly in Southeast Brazil, represent a frequently reported OPV zoonosis. Over the last decade, several VACV strains have been isolated from these outbreaks, also known as bovine vaccinia (BV) disease, and biological and molecular studies have shown a high degree of polymorphism among these isolates [15]. During BV outbreaks, cows exhibit lesions on the teats and udders, ranging from roseolar erythema to papules, vesicles, pustules, and crusts [16], [17]. Secondary bacterial mastitis is frequently associated with decrease in milk production, leading to economic losses and social impact, mainly in subsistence farming properties [18]. Infected milkers usually present lesions on their hands, apparently transmitted by unprotected contact with infected cattle [13], [19]. On some properties, the milking is performed without strict aseptic measures and the unsophisticated infrastructure of some farms allows for contact of cattle with wildlife and other domestic animals [18] such as small ruminants, dogs, cats and rodents.

The circulation of VACV within and/or among farms during BV outbreaks is usually promoted by infected milkers – who spread the virus to the herd by contact with their hands – and by the trade of infected cattle between properties [19]. However, some VACV outbreaks are temporally and spatially distant from previously notified BV areas. Therefore, the focal origin of outbreaks is frequently unknown. Moreover, BV usually occurs during the dry season, when some Brazilian biomes present a scarcity of victuals, leading some wild species to search for food in farm storehouse and corrals. Rats, mice, opossums, foxes, wild dogs and small felids are frequently observed around farming properties [20], [21]. In theory, some of these species, especially rodents, could be VACV reservoirs. In fact, at least three different VACV strains were isolated in Brazilian forests in the past, away from rural environments: Cotia virus (CTV)/SPAn 232 virus (SAV) was isolated from sentinel mice in São Paulo state [22], [23]; and BeAn 58058 virus (BAV) was isolated from the blood of an Amazon rodent specimen belonging to the *Oryzomys* genus [24]. Serological studies using sera from wild animals captured in the Brazilian Cerrado (a savanna-like environment) and animals from the Amazon biome have revealed a high prevalence of OPV-seropositive mammalians [25], [26]. Despite all the speculation about VACV circulation in Brazilian forests, the link between wildlife and BV outbreaks has not yet been established.

To date, the discovery of potential VACV hosts and reservoirs, as well as the study of viral circulation between wildlife and farms, could help to predict and perhaps prevent future BV outbreaks. In this study, we actively searched for VACV-infected peridomestic rodents (PdR) in BV-affected areas in Brazil and speculated about their role in outbreaks. We describe for the first time the isolation of VACV from a mouse during a BV outbreak. Our data show that this isolate was the same etiological agent of exanthematic lesions observed in the cattle and human inhabitants of a BV-affected area. These results provide new and important epidemiological information on VACV and lead to an interesting question: could PdRs be the link between wildlife and BV outbreaks?

## Results

### Ecological and epidemiological data

**Figure 1:**
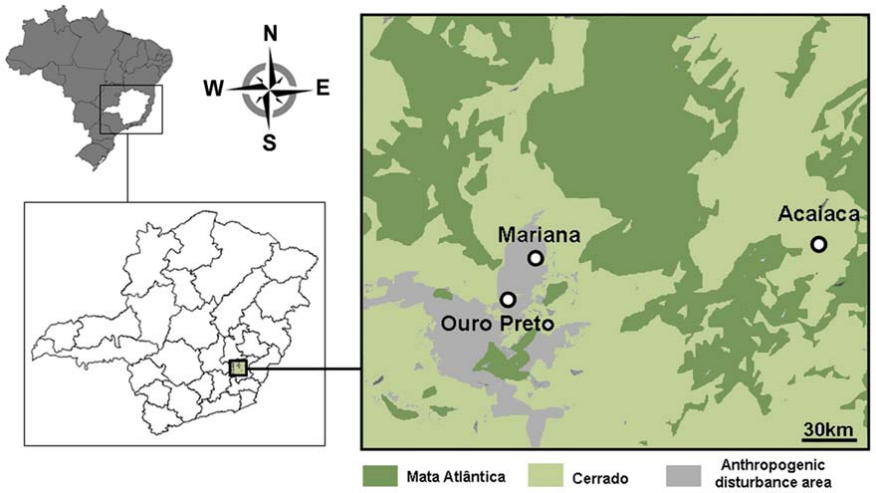
Location and phytoecological characteristics of Mariana county BV outbreak area. The outbreak region is located in a biome transition area, between the Mata Atlântica (Atlantic rainforest) and the Cerrado (Savanna), with accentuated anthropogenic disturbance.

Our investigation was performed in a BV outbreak-affected area around Mariana County (20°22′40″S - 43°24′57″W), in Minas Gerais State, Brazil (Figure 1). The region is localized in a biome transition area, between the Mata Atlântica (a tropical rainforest biome on the Brazilian Atlantic coast) and the Cerrado, which features fragments of deciduous seasonal forest alternated with savannas. The region's altitude is approximately 720 m, and it has a subtropical climate with average temperatures between 15–18°C. The outbreak occurred during the dry season, which generally lasts four months. The region presents accentuated anthropogenic disturbances, with several small plantations (sugar cane, coffee and corn) and livestock areas. Debris and garbage could be observed in the BV-affected properties and PdRs, and their excrements were also observed. In some cases, portions of forest marked the limit of the backyards, corrals and pastures, and wild animals including mice, rats, little spotted cats, capybaras, red-legged siriemas, opossums, marmosets and cattle egrets were frequently sighted around the properties.

Exanthematic infections were notified in several small properties localized within the analyzed area. No outbreak had ever been declared in this region previously. Typical VACV lesions were observed on cattle's teats and on milkers' hands and arms. Although serological and molecular tests confirmed human and bovine infection by VACV, other farm animals, such as dogs, pigs, cats and horses were OPV-seronegative based on plaque reduction neutralizing tests (PRNT).

### Viral isolation from a peridomestic mouse

A total of six house rats (*Rattus rattus*) and seven house mice (*Mus musculus*) were trapped in different BV-affected properties. They were identified according to morphological characteristics using the Brazilian Rodents Guide and taxonomical keys (Pan-American Health Organization, 2008), and by mastozoologists from the Zoology Department of our institute. To data, both *Rattus rattus* and *Mus musculus* are Old World species, artificially introduced in Brazil. The rodents were captured in the vicinity of the farms, including in corrals, food warehouses, grain plantations and boundaries of adjacent forests.

Although none of the rodents presented apparent VACV epidermal lesions or morphological changes in collected organs, neutralizing antibodies against OPV were detected in two serum samples collected from adult mice captured close to a small cornfield, with titers of 1∶40 and 1∶320 neutralizing units/ml. The inoculation with clarified material from the peritoneum and testicles of the mouse with the highest level of OPV-neutralizing antibodies in chicken embryo fibroblast (CEF) monolayers induced the formation of typical poxvirus cytopathic effects after 72 hours. This isolate, named Mariana virus (MARV), was further multiplied in BSC-40 cells and purified in order to perform biological and molecular characterizations.

### Viral biological and phylogenetic analysis

**Figure 2:**
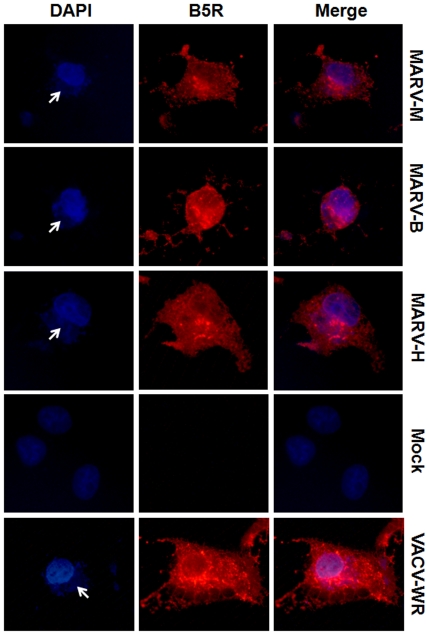
Immunofluorescence assay of infected BSC-40 cells revealed that MARV-M, MARV-H and MARV-B are OPV. BSC-40 cells were infected with MARV isolates at a MOI of 3. After 24 h, an anti-OPV antibody revealed the presence of the B5R protein which was punctually distributed, especially in cytoplasmic membranes and the Golgi complex, similarly to the pattern observed in VACV-WR-infected cells. The DAPI staining highlights the DNA contained in viral factories (white arrows).

**Figure 3:**
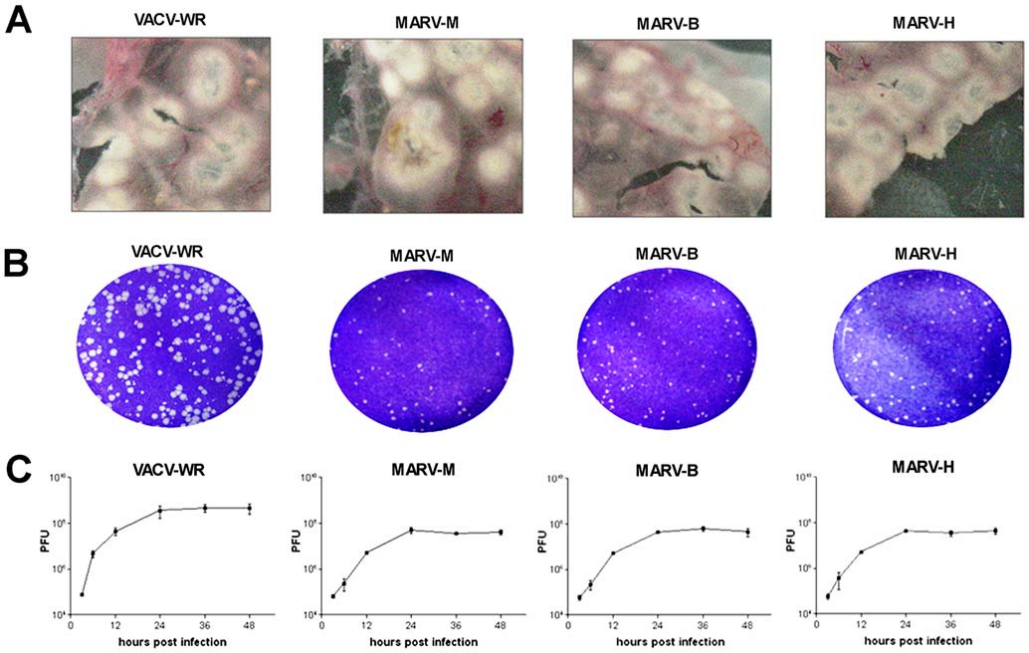
MARV-M, MARV-B and MARV-H induced the formation of white pocks in CAM and presented a similar profile in plaque phenotype and one-step growth curve assays. (A) CEF monolayers infected with MARV isolates were harvested after 72 h and inoculated onto the CAM of embryonated chicken eggs. Typical VACV white pocks were observed in inoculated CAM after 72 hours. (B) MARV isolates presented small plaques in BSC-40 cells (average diameter: 0.6 mm), distinct from the prototypical VACV-WR plaque phenotype (average diameter: 1.4 mm). (C) One-step growth curve assays in BSC-40 cells showed that MARV-B, MARV-H and MARV-M present a similar replication profile, which was statically distinct from that observed for VACV-WR (p<0.04).

In order to investigate whether MARV was, in fact, the etiological agent of the local BV outbreak, viruses were also isolated from a human vesicle and from a bovine scab, and used for biological and molecular comparisons. The murine isolate was designated MARV-M; the bovine, MARV-B; and the human, MARV-H. Immunofluorescence microscopy from BSC-40 cells infected with MARV isolates (Figure 2) revealed the presence of the OPV B5R protein which was punctually distributed, especially in the cytoplasmic membranes and the Golgi complex, similarly to the pattern observed in VACV-WR infected cells. The DAPI staining highlighted the DNA contained in viral factories (white arrows). The inoculation of MARV isolates in the chorioallantoic membrane (CAM) of embryonated chicken eggs caused the appearance of white pocks, indicative of VACV infection, as seen in Figure 3-A. No difference was observed between MARV isolates and VACV-WR in the immunofluorescence and CAM inoculation assays.

One-step growth curve assays in BSC-40 cells showed that MARV-B, MARV-H and MARV-M presented a similar replication profile, but statistically distinct from that observed in VACV-WR infected cells (*p*<0.04) (Figure 3-C). While MARV isolates presented a growth rate of approximately 1.5 log, VACV-WR titers increased 2.0 log after 24 hours of infection. Additionally, plaque phenotype comparisons showed that MARV isolates induced the formation of small plaques in BSC-40 cells (average diameter: 0.6 mm), different from the large plaques formed by VACV-WR (average diameter: 1.4 mm) (Figure 3-B).

**Figure 4:**
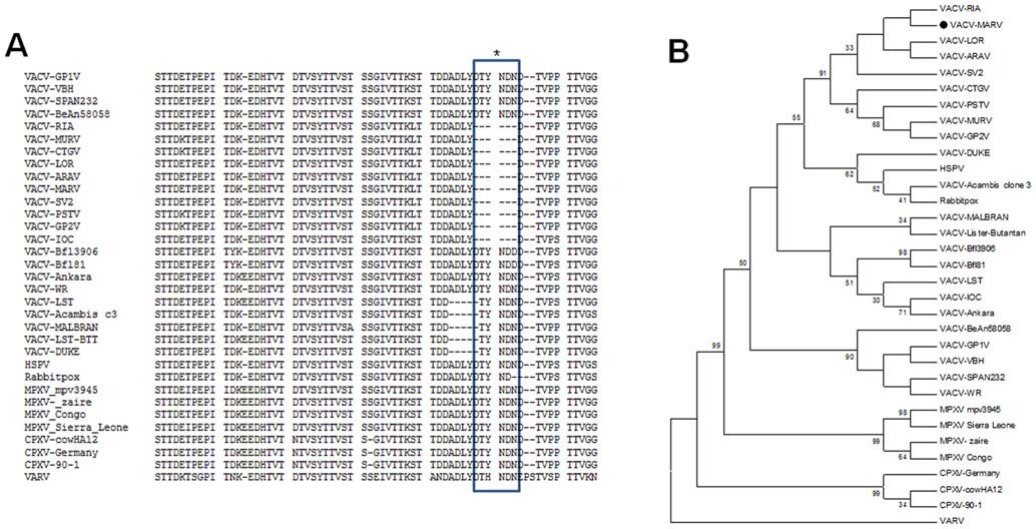
MARV is grouped phylogenetically with several Brazilian VACV strains isolated during BV outbreaks, but is segregated from VACV vaccine strains. (A) Amino acid inferred sequence of the MARV hemagglutinin (HA) gene and comparison to the homologous sequence from several OPV. The box with an asterisk indicates the deletion region conserved in the sequences of MARV, several Brazilian field VACV samples and the IOC vaccine strain. (B) Phylogenetic tree constructed based on the nucleotide sequence of the OPV ha gene. The *ha* tree shows MARV grouping with VACV field samples. Despite the presence of the 6 aa deletion, the IOC strain grouped with other vaccine strains. Nucleotide sequences were obtained from GenBank (VACV-ARAV:AY523994; VACV-LOR:DQ810281; VACV-RIA:DQ810280; VACV-CTGV:AF229247; VACV-PSTV:DQ070848; VACV-MURV:DQ247770; VACV-GP2V:DQ206437; VACV-Acambis clone3:AY313848; VACV-DUKE:DQ439815; VACV-MALBRAN:AY146624; VACV-SV2:EF063677; Rabbitpox:AF37511; VACV-LST:AY678276; VACV-SPAn232:DQ222922; VACV-BeAn58058:DQ206442; VACV-GP1V:DQ206436; VACV-WR: AY243312; VACV-Ankara:U944848; VACV-VBH:DQ206435; VACV-Lister-Butantan:EF175985; VACV-IOC:AF225248; VACV-Bfl3906:AF375077; VACV-Bfl81:AF375078; HSPV:DQ792504; MPXV-ZAIRE:DQ011155; MPXV-mpv3945:AF375098; MPXV-Sierra Leone:AY741551; CPXV-Germany: DQ437593; CPXV-cowHA12:AY902253; CPXV-GRI90:AF375087; VARV:DQ437589. Bootstrap confidence intervals are shown on branches (1,000 replicates).

OPV *timidine kinase* (*tk*), *virus growth factor* (vgf) and *hemmaglutinin* (*ha*) genes from MARV isolates were submitted to PCR amplification, generating 528-, 381- and 960-bp fragments, respectively. Amplicons were directly sequenced in both orientations. The alignment of the *tk*, *vgf* and *ha* sequences obtained from MARV-M, MARV-B and MARV-H displayed perfect homology; therefore, all isolates were considered identical (MARV) according to phylogenetic parameters. When compared to nucleotide sequences available in the GenBank databases using the BLASTN program, the *tk* and *vgf* genes from MARV were highly similar to homologous genes from other VACV strains. Optimal alignment showed similarity rates of up to 99.5% between MARV and VACV–WR genes and minimal differences from nucleic acid substitutions. The coding region of the *ha* gene was analyzed by alignment with similar sequences from other OPV isolates deposited in GenBank. The MARV *ha* inferred amino acid sequence contained a signature deletion (Figure 4-A) also present in the sequences of other Brazilian VACV strains, such as Araçatuba virus (ARAV), Cantagalo virus (CTGV), Serro virus (SV2), GuaraniP2 virus (GP2V), Muriae virus (MURV) and others, but absent in GuaraniP1 virus (GP1V), Belo Horizonte virus (VBH), BeAn58058 virus (BAV) and SPAn232 virus (SAV). Although the deletion is also present in the VACV-Instituto Oswaldo Cruz (IOC) strain, other vaccine strains, including Lister-Butantan and Malbran, the vaccine strains used in Brazil during the WHO campaign, lacked this signature [1]. Based on the *ha* nucleotide sequences from MARV and other OPV, we constructed an evolutionary tree employing the MEGA 3.1 program, which clustered MARV with several VACV isolates (Figure 4-B).

## Discussion

The present study introduces a new element in the complex and poorly understood VACV transmission chain: the peridomestic rodent. The search for VACV-infected rodents in BV-affected areas seemed like a rational strategy, since (i) such animals have been described as CPXV reservoirs in Europe, promoting viral transmission to humans, cats and zoo animals [6]; (ii) rats and mice are frequently sighted in BV-affected farms and are in constant contact with wildlife, humans and farm animals [20], [21]; and (iii) laboratory studies showed that PdRs can shed and transmit OPV by direct contact with contaminated excrement [27], [28]. Although some VACV strains had consistently been isolated and re-isolated from Brazilian forests using mice as sentinel animals [22], we describe for the first time the isolation of a VACV strain from a *Mus musculus* mouse captured in the context of a BV outbreak. Previous phylogenetic analysis of conserved and variable OPV genes showed that some rodent/forest VACV isolates cluster with specific zoonotic/bovine VACV isolates [13], [14]. However, the present work is the first to clearly establish a link between a human-, a bovine- and a murine-VACV isolates that are temporally and spatially related.

Epidemiological and phylogenetic investigations of OPV have been targeting the *ha* nucleotide and amino acid sequences to identify viral species and estimate their evolutionary relationships [16], [29], [30]. Recently, rat-to-elephant and rat-to-human CPXV transmission were described [29], [30], on the basis of the perfect homology of the *ha* gene open reading frame (ORF) of CPXV isolates from distinct hosts. Therefore, based on this model, we decided to analyze the *ha* gene and the *vgf* and *tk* conserved genes in parallel with biological assays to characterize and compare the murine, bovine and human MARV isolates. The analysis of *tk*, *vgf* and *ha* sequences from MARV isolates displayed perfect homology not only in their respective ORFs but also in the entire nucleotide sequences analyzed. The genetic similarity observed among these samples was corroborated by results obtained in one-step curve and plaque phenotype assays, indicating that the three isolates were in fact from a single VACV strain that had circulated among different hosts during the BV outbreak in Mariana County.

Trindade et al. [13] and Drumond et al. [14] demonstrated that there are two genetically distinct VACV groups circulating in Brazil. The first is composed of ARAV, CTGV, GP2V, SV2, PSTV and other strains, while group 2 includes BAV, SAV, GP1V and VBH strains. Interestingly, this genetic polymorphism is reflected in the VACV strains' virulence profiles in Balb/c mouse models [31]. Our analysis of the *ha* gene revealed this phylogenetic segregation pattern, and MARV clustered with the first group. All VACV strains from group 1 presented a deletion of 6 amino acids at position 251, but not all isolates that present such a deletion were clustered in this group. VACV-IOC, for example, despite presenting the deletion, was grouped with VACV-Ankara and VACV-Lister, both vaccine strains. Therefore, our data suggest that MARV shares the same probable origin as many Brazilian VACV strains isolated during BV outbreaks, and it is distinct from the vaccine strains used in Brazil during the smallpox eradication campaign, such as LST-BTT, Malbran and IOC [1].

The occurrence of VACV circulation among wild animals was supported by viral isolations in the 1960s and 1970s [22]–[24] and by recent serological studies that showed a high prevalence of anti-OPV immunoglobulins from mammalians in Brazilian wildlife, including rodents, macaques, grey-foxes and coatis [25], [26]. As mentioned, some BV outbreaks were temporally and spatially distant from other outbreaks, in areas with little or no pecuary activity. In these specific cases, it is believed that wildlife was the original source of VACV circulation. Viruses could then reach rural environments when their wild hosts eventually leave their wild habitat and venture to the boundaries of farms in search of food (mainly in plantations) or after the deforestation of their habitat. Nonetheless, the presence of wild animals is usually restricted to the properties' borders, with their presence in places like corrals and the main house being quite atypical [31]. Only wolves, foxes and opossums have been seen in peridomestic buildings. Therefore, if these VACV strains are indeed primarily circulating among wild mammals, the isolation of MARV from a peridomestic mouse could represent a possible link between wildlife and BV-affected properties, as PdRs usually wander into the farms and their surroundings, maintaining contact with humans, cattle and wild animals alike [31].

**Figure 5:**
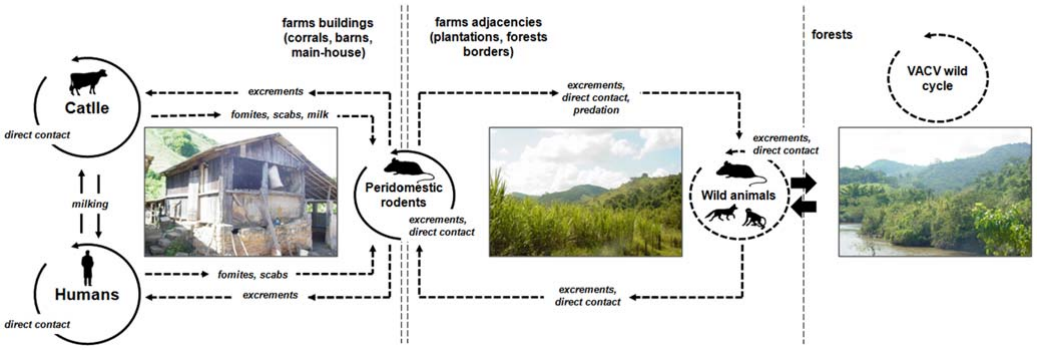
A hypothetical model of the VACV transmission cycle. Peridomestic rodents could promote the transmission of VACV between wild animals and cattle or humans, since they circulate both in farm buildings and their surroundings. This diagram was proposed based on published epidemiological and laboratory data on VACV and the behavioral characteristics of Brazilian rodents and wild animals. Solid lines indicate experimentally determined data, and dashed lines represent hypothetical propositions still under investigation.

Based on our data and on previously published works [16], [18], [19], [27], including epidemiological studies of other OPV [12], [28], [29], we developed the hypothetical VACV transmission cycle presented in Figure 5. PdRs could be infected by wild animals in the farms' surroundings after territorial fights [32], ingestion or aspiration of contaminated feces [27], consumption of contaminated carcasses [12] or consumption of food containing the saliva of an infected animal [12]. VACV and CPXV shedding and transmission have been consistently demonstrated in mice and rats [27], [28], which can partially explain the circulation and spread of VACV among PdRs. Some infected PdRs eventually return to the farms, introducing VACV into bovine and human populations. On the other hand, peridomestic mice and rats could be infected with VACV after coming into contact with bovine/human scab fragments, contaminated milk (Abrahão & Oliveira et al., unpublished data) or fomites (bulk tanks, cloth contaminated with blood and tissue from bovine lesions and materials associated with milking procedures). Subsequently, infected PdRs could spread VACV to wild animals when they are predated upon [1], via their feces and carcasses or during fights. Although less probable, the possibility of direct VACV transmission between wild animals and humans/cattle cannot be discarded. Additional studies are required to elucidate the complex VACV transmission dynamics, especially when considering the virus's large host range. We believe that the presence of VACV-infected PdRs in affected properties could represent both the cause and/or the consequence of BV outbreaks. Monitoring of PdRs could be used to predict BV outbreaks or to indicate the risk of VACV introduction into wild environments.

## Materials and Methods

### Clinical sample collection and rodent capture

Samples of vesicles and crusts (dried scabs) from cattle teats and milkers' hands were collected during a BV outbreak in Mariana County, Brazil (2005), using 1 ml insulin syringes and 0.45 mm×13 mm needles, cotton swabs or a pair of tweezers. Serum samples were collected from milkers, cows and other domestic animals, such as dogs, cats, pigs and horses and used in neutralization assays [33]. A written consent was provided by the inhabitants of the analyzed rural properties before participating in this study. During the same outbreak, a total of 20 slide-door traps for rodents were assembled using a mixture of cod liver oil, peanut butter, banana and bacon as bait. Traps were positioned at several points in and around the farms, including in barns, silos, corrals, food warehouses, grain plantations and the boundaries of adjacent forests. The captured rodents were anesthetized for blood collection and subsequently sacrificed by cervical dislocation. The brain, liver, lungs, heart, gonads, intestine, trachea, peritoneum, stomach and spleen were collected and stored until viral isolation experiments could be conducted. The rodent captures were performed with the written permission of the Brazilian Environmental Surveillance Institute (IBAMA), and all experiments were approved by the Ethical and Animals Use Committee at the Universidade Federal de Minas Gerais, Brazil.

### Cells and viruses

Chicken embryo fibroblasts (CEF) and BSC-40 cells were grown at 37°C in Eagle's minimum essential medium (MEM; GibcoBRL, Invitrogen, Carlsbad, California, USA) supplemented with 5% fetal calf serum (FCS, Cultilab, Brazil), 25 µg/ml fungizone (Amphotericin B, Cristália, São Paulo, Brazil), 500 U/ml penicillin and 50 µg/ml gentamicin (Schering-Plough, Brazil). The VACV strain *Western Reserve* (VACV-WR), kindly provided by Dr C. Jungwirth (Universitat Wurzburg, Germany), and the VACV-MARV isolates were grown in BSC-40 cells and purified in a sucrose gradient as previously described [34].

### Sample preparation and viral isolation

Scabs and murine organs were macerated in phosphate buffered saline (PBS) (0.1 g sample/0.9 ml PBS) using a homogenizer (Politron, Switzerland) and clarified by centrifugation at 2000×g for 3 minutes. Vesicular liquid swabs were added to 200 µL of PBS and centrifuged at 2000×g for 3 min. Viral isolation was performed by inoculation of 200 µL of processed samples onto CEF monolayers, followed by 72 hours of incubation. Monolayers presenting cytopathic effects were harvested and inoculated onto the chorioallantoic membrane (CAM) of embryonated chicken eggs and incubated at 37°C for 72 hours to analyze the appearance of typical VACV white pocks [35].

### Immunofluorescence microscopy

BSC-40 cells were grown to a density of 4.5×10^4^ cells on sterile 13 mm coverslips and infected with MARV isolates or VACV-WR at a multiplicity of infection (MOI) of 3. After 24 hours, the cells were fixed with 4% paraformaldehyde solution and incubated with an anti-OPV B5R antibody, followed by incubation with secondary rhodamine-conjugated anti-mouse antibody. After incubation with antibodies, cells were stained with DAPI for 10 minutes. Images of fluorescently-labeled cells were obtained with an Olympus BX51 immunofluorescence microscope.

### Viral infectivity assays

BSC-40 cells were grown to a density of 5×10^5^ cells per well on a 6-well culture dish and then infected with MARV isolates and VACV-WR. Infections were carried out at a MOI of 10 for 3, 6, 12, 24, 36 and 48 h. The infected monolayers were then harvested and titrated in BSC-40 cells for one-step growth curve assays. MARV isolates and VACV-WR plaque phenotype assays were carried out in parallel, in BSC-40 cells at 0.01 MOI and incubated for 48 h. Statistical analysis using the two-tailed Student's T-test was performed with ABI prism 3.0 software.

### Amplification of *tk*, *vgf* and *ha* genes and phylogenetic analyses

Primers based on the *tk* and *vgf* nucleotide sequence of VACV–WR were produced as described by Fonseca et al. [24]. The *ha* coding sequence was amplified using primers EACP1 and EACP2, as described by Ropp et al. [36]. The MARV PCR-amplified *tk*, *vgf* and *ha* fragments were directly sequenced in both orientations and in triplicate (Mega-BACE sequencer, GE Healthcare, Buckinghamshire, UK). The sequences were aligned with previously published OPV sequences from GenBank using the ClustalW method, and were manually aligned using MEGA software version 3.1 (Arizona State University, Phoenix, AZ, USA). The Modeltest software was used determine which model of evolution was most appropriate for our analysis [37]. A phylogenetic tree based on the *ha* gene sequence was constructed using the maximum parsimony method with 1,000 bootstrap replicates implemented by MEGA 3.1. The MARV *tk* (**GQ226042**), *vgf* (**GQ226041**) and *ha* (**GQ226040**) sequences were deposited in GenBank.
